# Quantitative *T*_2_ mapping MRI links dental caries severity to histological pulp degeneration in human teeth

**DOI:** 10.17305/bb.2026.14331

**Published:** 2026-06-29

**Authors:** Ana Tenyi, Aleksandra Milutinović, Ksenija Cankar

**Affiliations:** 1 Department of Dental Diseases and Normal Dental Morphology, Faculty of Medicine, University of Ljubljana, Ljubljana, Slovenia; 2 Institute of Histology and Embryology, Faculty of Medicine, University of Ljubljana, Ljubljana, Slovenia; 3 Institute of Physiology, Faculty of Medicine, University of Ljubljana, Ljubljana, Slovenia

**Keywords:** Dental MRI, T2 mapping, dental pulp histology, pulp degeneration, caries severity

## Abstract

Advancing carious lesions may induce structural and degenerative changes within the dental pulp that are not directly captured by conventional diagnostic methods. This study aimed to evaluate the association between caries severity, quantitative magnetic resonance imaging (MRI) *T*_2_ mapping, and histological pulp degeneration (PD). Fifty-two permanent premolars scheduled for orthodontic extraction were clinically assessed using the International Caries Detection and Assessment System (ICDAS) and classified as ICDAS 0–3. Before extraction, participants underwent 3-tesla (3T) MRI using a multi-echo spin-echo *T*_2_ mapping protocol. Mean *T*_2_ relaxation times and *T*_2_ variability, defined as the standard deviation of *T*_2_ values, were calculated within manually delineated coronal pulp regions of interest (ROIs). After extraction, pulp tissues were processed for hematoxylin and eosin staining, and PD was graded on a 0–3 scale and quantified morphometrically as the proportion of affected pulp area. Histological PD was identified in 12 of 52 teeth and was observed only in carious teeth with ICDAS 2–3 lesions. Teeth with PD showed lower mean *T*_2_ relaxation times than teeth without PD (114.236 ± 28.020 ms vs. 131.740 ± 23.562 ms; *P ═* 0.049) and lower *T*_2_ variability (33.226 ± 7.080 ms vs. 41.463 ± 8.366 ms; *P ═* 0.006). PD grade correlated positively with ICDAS score (*ρ ═* 0.481; *P ═* 0.00035) and negatively with mean *T*_2_ relaxation time (*ρ ═* −0.367; *P ═* 0.00896) and *T*_2_ variability (*ρ ═* −0.381; *P ═* 0.00708). Greater morphometric PD area was also associated with lower mean *T*_2_ relaxation time and *T*_2_ variability. These findings suggest that caries severity is associated with histological pulp degeneration and reduced MRI-derived *T*_2_ parameters, supporting quantitative *T*_2_ mapping as a noninvasive approach for investigating caries-related pulpal tissue changes.

## Introduction

Extension of carious lesions toward the dental pulp represents a critical stage in the disease process, directly influencing treatment planning and long-term tooth prognosis. The extent of these lesions is typically assessed using clinical scoring systems, such as the International Caries Detection and Assessment System (ICDAS), which provides a standardized evaluation based on the tooth’s surface characteristics [1]. However, this surface-level assessment does not offer insights into the underlying biological condition or the extent of pulpal damage. In routine clinical practice, pulpal status is indirectly evaluated through sensibility tests, which rely on neural responses or structural changes rather than direct assessment of pulpal tissue health, requiring clinicians to infer the underlying condition [2]. Furthermore, radiographic examinations only visualize dental hard tissues. Consequently, significant uncertainty often exists regarding the pulp’s true biological response to carious insult. This diagnostic gap has spurred interest in imaging techniques capable of more directly assessing pulpal tissue, with magnetic resonance imaging (MRI) emerging as a promising non-invasive approach [3–5].

Unlike conventional radiographic methods, MRI enables soft tissue visualization without ionizing radiation. Recent advancements in dental MRI allow for *in vivo* assessment of pulpal morphology and tissue characteristics [3, 6, 7]. *T*_2_ mapping, in particular, has garnered attention because *T*_2_ relaxation times are influenced by tissue hydration, extracellular matrix organization, and inflammatory changes [4]. These factors are central to the pulpal response to carious irritation, suggesting that *T*_2_ mapping can capture biologically relevant changes undetectable by standard diagnostic tools [8].

*T*_2_ mapping provides a quantitative assessment of both the average relaxation time of the pulp tissue and its signal variability, typically expressed as the standard deviation (SD) of *T*_2_ relaxation times. This measure reflects tissue heterogeneity, capturing differences in water content, cellular density, and microstructural organization, including subtle or localized changes that might be overlooked when considering only mean values [9, 10]. By integrating both mean *T*_2_ relaxation times and their variability, MRI offers a more nuanced assessment of pulpal tissue integrity. This approach provides insight not only into the overall status of the pulp tissue but also into the spatial distribution of degenerative or inflammatory changes that may occur in response to carious lesions [10].

Reliable assessment of pulpal status is crucial for selecting appropriate treatment strategies. However, standard clinical tests provide limited information about the pulp’s histological condition and may not accurately reflect the extent or reversibility of tissue changes [11]. This often leads to clinical decisions made without precise biological information. Imaging modalities capable of more directly reflecting pulpal tissue alterations could therefore enhance diagnostic accuracy and support more individualized treatment planning [12, 13]. Quantitative MRI techniques have demonstrated promise in linking imaging findings with pulpal tissue responses, indicating their potential value in this context [3, 5, 6, 10].

Histological examination of caries-affected pulp frequently reveals degenerative changes alongside inflammatory responses. Our previous study demonstrated increased inflammatory cell infiltration within the dental pulp even in teeth with ICDAS 1 lesions, indicating that pulpal responses can occur during the earliest stages of caries progression [10]. Chronic inflammation is known to increase vascular permeability through inflammatory mediators released by immune cells, leading to tissue edema. Because the dental pulp is enclosed within rigid mineralized walls, even a relatively small increase in tissue fluid can compromise microcirculation and contribute to localized hypoxic stress. Prolonged hypoxia and chronic inflammatory stimulation have been associated with structural alterations of the pulp tissue, including degenerative changes. Similar mechanisms have been proposed in studies investigating pulpal degeneration associated with chronic periodontal inflammation, where increased expression of hypoxia-related markers such as hypoxia-inducible factor 1-alpha (HIF-1α) has been reported [14]. Collectively, these observations suggest a possible biological pathway linking early pulpal inflammatory responses with subsequent degenerative alterations. Therefore, inflammatory and degenerative changes within the pulp may coexist, and degenerative alterations may be observed even in teeth with relatively early stages of caries development.

Pulp degeneration (PD) is characterized by the formation of vacuolated areas within the pulp tissue and may be associated with prolonged or low-grade irritation [15]. Although PD has been repeatedly described in histopathological studies [14, 16], it has received limited focused investigation. Its extent, grading, and possible clinical implications remain insufficiently defined, and quantitative correlations with diagnostic findings are largely absent from the literature.

Crucially, the relationship between MRI pulp tissue evaluation and specific degenerative features of the dental pulp has not yet been clarified. While previous research has explored associations between MRI findings and general pulpal changes, distinct patterns of degeneration, such as PD, have not been systematically examined in relation to MRI imaging biomarkers.

The present study aimed to assess caries severity using the ICDAS system, quantitatively evaluate pulpal tissue characteristics using magnetic resonance (MR) *T*_2_ mapping, and correlate imaging results with histological grading of PD. By integrating clinical assessment, advanced imaging, and histological evaluation, this study seeks to contribute to a more biologically grounded understanding of pulpal changes associated with caries and to further explore the diagnostic potential of MRI in dentistry.

**Figure 1.**
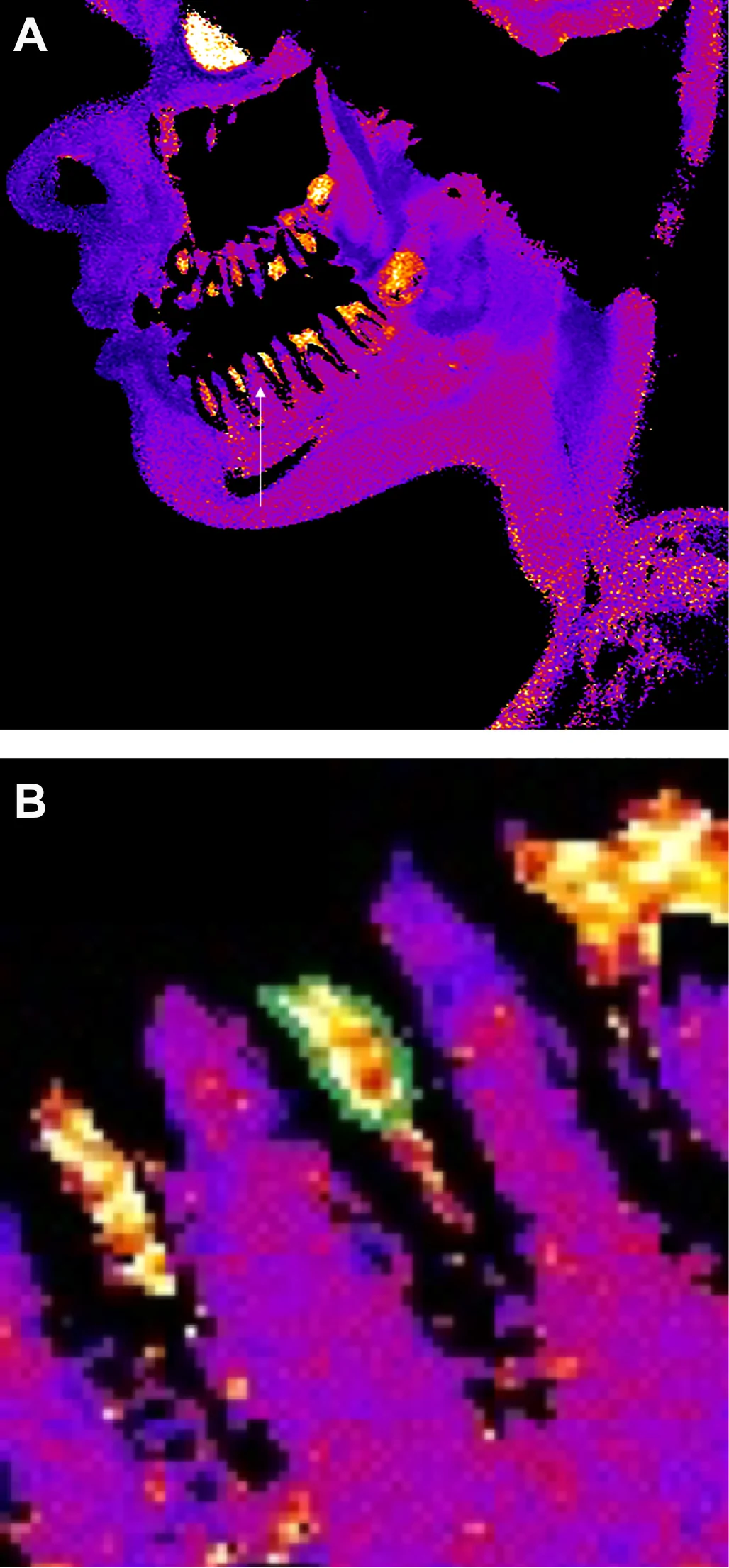
**Representative sagittal MRI-derived *T*_2_ map used for coronal pulp analysis.** (A) Sagittal color-coded *T*_2_ map showing the examined tooth, indicated by the white arrow. (B) Enlarged view of the corresponding coronal pulp region. The manually delineated region of interest, outlined in green, was restricted to the pulp tissue and excluded the surrounding dentin to minimize partial volume effects. For the illustrated specimen, the mean *T*_2_ relaxation time was 136.15 ms, and *T*_2_ variability, calculated as the SD of *T*_2_ values within the ROI, was 39.85 ms. **Abbreviations**: MRI, magnetic resonance imaging; ms, milliseconds; ROI, region of interest; SD, standard deviation.

## Materials and methods

### Patients and tooth selection

This study was approved by the Slovenian Medical Ethics Committee (Approval No. 0120–415/2020/6). Written informed consent was obtained from all participants prior to inclusion. Premolars scheduled for extraction due to orthodontic crowding were selected. Teeth were required to be free of restorations, cracks, or previous endodontic treatment. Clinical caries status was assessed prior to extraction using the ICDAS.

### Clinical caries assessment

A total of fifty-two upper and lower permanent premolars were included in the study, obtained from 14 patients aged 12–20 years. Prior to extraction, each patient underwent a clinical examination, during which caries status was evaluated on all tooth surfaces using the ICDAS [1]. Teeth were cleaned and dried before ICDAS assessment and evaluated according to standardized ICDAS criteria by a trained examiner following previously established protocols [1]. Formal inter- and intra-examiner reliability analyses were not performed.

Based on the recorded ICDAS scores, teeth were categorized into four groups: Group A (sound teeth, ICDAS score 0); Group B (initial carious changes, ICDAS score 1); Group C (initial carious lesions, ICDAS score 2); and Group D (carious lesions with visible and tactile enamel cavitation, ICDAS score 3). For tooth-level analyses, the ICDAS score assigned to each tooth corresponded to the highest ICDAS score observed on any evaluated tooth surface.

### MRI

The study cohort and MRI acquisition protocol have been previously described in Tenyi et al., 2024 (BMC Oral Health) [10]. The previous study focused on the association between ICDAS-based caries severity, whole-pulp MRI-derived *T*_2_ relaxation times, and inflammatory infiltrate characterization within the dental pulp. In the present investigation, the same cohort was reanalyzed to address a distinct research objective, specifically focusing on histological PD, including its grading and morphometric quantification, and its correlation with quantitative *T*_2_ mapping parameters. Additionally, MRI analysis in the current study was restricted to the coronal pulp region to reduce potential partial volume effects from adjacent dentin tissues with very low *T*_2_ values.

All participants underwent MRI prior to tooth extraction. MRI examinations were performed using a clinical MRI system equipped with a 32-channel head coil. A quantitative *T*_2_ mapping protocol was applied to assess pulpal tissue characteristics. Imaging parameters, including echo times, repetition times, slice thickness, and field of view, were selected to optimize visualization of the dental pulp and were consistent across all examinations.

*T*_2_ maps were generated using a multi-echo spin-echo (MESE) sequence, and voxel-wise *T*_2_ relaxation times were calculated via mono-exponential fitting. Regions of interest (ROIs) were manually delineated within the coronal pulp tissue on the generated *T*_2_ maps by an experienced assessor, blinded to histological findings, to avoid partial volume effects from surrounding dentin. Mean *T*_2_ relaxation times were calculated for each tooth and utilized in subsequent analysis (Figure 1).

### MR image acquisition

MRI was conducted using a 3-T whole-body MRI system (TX Achieva, Philips, Netherlands) equipped with an 80 mT/m maximum gradient strength. A 32-channel receive head coil was used for all examinations. MRI scans were performed on the same day as the clinical assessment. Prior to imaging, all participants maintained their usual daily activities without restriction.

The imaging protocol commenced with moderate spatial resolution images to localize dental pulp anatomy. Quantitative *T*_2_ mapping was subsequently performed using MESE *T*_2_-weighted sequence acquired in sagittal orientation, with the field of view adjusted to encompass the majority of the pulp chamber. The MESE sequence parameters were: repetition time/echo time (TR/TE) of 2000/15 ms, with additional echo times at 30, 45, 60, 75, and 90 ms; field of view of 160 × 160 mm^2^; slice thickness of 2.5 mm; acquisition and reconstruction matrices of 380 × 311 and 560 × 560, respectively; acquisition and reconstruction voxel sizes of 0.42 × 0.51 × 2.5 mm^3^ and 0.29 × 0.29 × 2.5 mm^3^, respectively; three slices with an interslice gap of 0.25; number of signal averages set to 1; and no signal acquisition acceleration. The total acquisition time for all six echoes was 10 min and 24 s [8, 10].

### MR data analysis

MESE images were processed to generate *T*_2_ relaxation time maps using a pixel-wise mono-exponential fitting approach with non-negative least-squares optimization. This analysis was performed using the MRI Analysis Calculator plugin in ImageJ software (National Institutes of Health, USA). The resulting calculations yielded quantitative *T*_2_ maps for each imaged slice.

To minimize noise, *T*_2_ relaxation times were averaged within predefined regions, excluding pixels with zero *T*_2_ values, as these corresponded to areas outside the pulp tissue. The resultant mean *T*_2_ relaxation times were used for subsequent statistical evaluation. *T*_2_ variability was calculated as the SD of *T*_2_ relaxation times within each ROI.

### Tooth preparation for pulp tissue samples

Immediately following extraction, approximately one-third of the apical portion of each root was removed to facilitate fixative penetration into the pulp tissue. Specimens were then immersed in formalin for 24 h.

After initial fixation, each tooth was longitudinally split along its vertical axis. The halves containing the pulp were returned to formalin for an additional 48 h to ensure complete fixation. The pulp tissue was subsequently carefully extracted from the tooth halves using a dental probe, dehydrated through a graded alcohol series, cleared in xylene, and embedded in paraffin. Serial longitudinal sections of 4.5 µm thickness were prepared with a step interval of 20 µm between consecutive sections. Sections were stored at room temperature until further processing [10, 17].

### Histological staining and evaluation

Histological sections were stained with hematoxylin and eosin (HE) for detailed visualization of pulpal tissue components. Particular attention was given to identifying PD, defined as the presence of pulp or vacuole-like spaces within the pulp tissue accompanied by disruption of normal tissue architecture. PD was evaluated using a semi-quantitative grading system. Representative sections were documented using light microscopy.

### PD grading protocol

Grading was performed by a single experienced histologist blinded to clinical and MRI findings. Histological grading was based on evaluating a representative histological section, with the final grade assigned according to the estimated overall proportion of the section affected by degenerative changes. The presence and extent of PD were assessed based on the appearance of reticular or vacuole-like changes within the pulp tissue and disruption of normal pulp architecture. Degenerative changes were identified only when vacuolated or reticular spaces were accompanied by reproducible structural alterations, including reduced cellularity, disruption of normal pulp architecture, and clear demarcation from adjacent unaffected tissue. A semi-quantitative scale from 0 to 3 was applied as follows:

**Grade 0:** No detectable PD; pulp tissue appears intact with normal cellular and extracellular organization.

**Grade 1:** Minimal pulp changes affecting less than 25% of the pulp; small vacuolated areas present, but overall tissue architecture largely preserved.

**Grade 2:** Moderate PD involving 25%–50% of the pulp; vacuolated areas are more widespread, with partial disruption of connective tissue organization.

**Grade 3:** Extensive PD affecting more than 50% of the pulp; large vacuolated areas, marked loss of normal pulp structure, and significant disruption of connective tissue architecture.

In cases demonstrating overlapping histological features between two adjacent degeneration grades, intermediate categories (e.g., 1–2 or 2–3) were assigned to more accurately reflect observed morphological characteristics. For statistical analyses, intermediate grades were coded as continuous values, with grade 1–2 assigned a value of 1.5 and grade 2–3 assigned a value of 2.5.

Representative micrographs were documented for reference, and grading consistency was confirmed by re-evaluating a random subset of sections.

**Figure 2.**
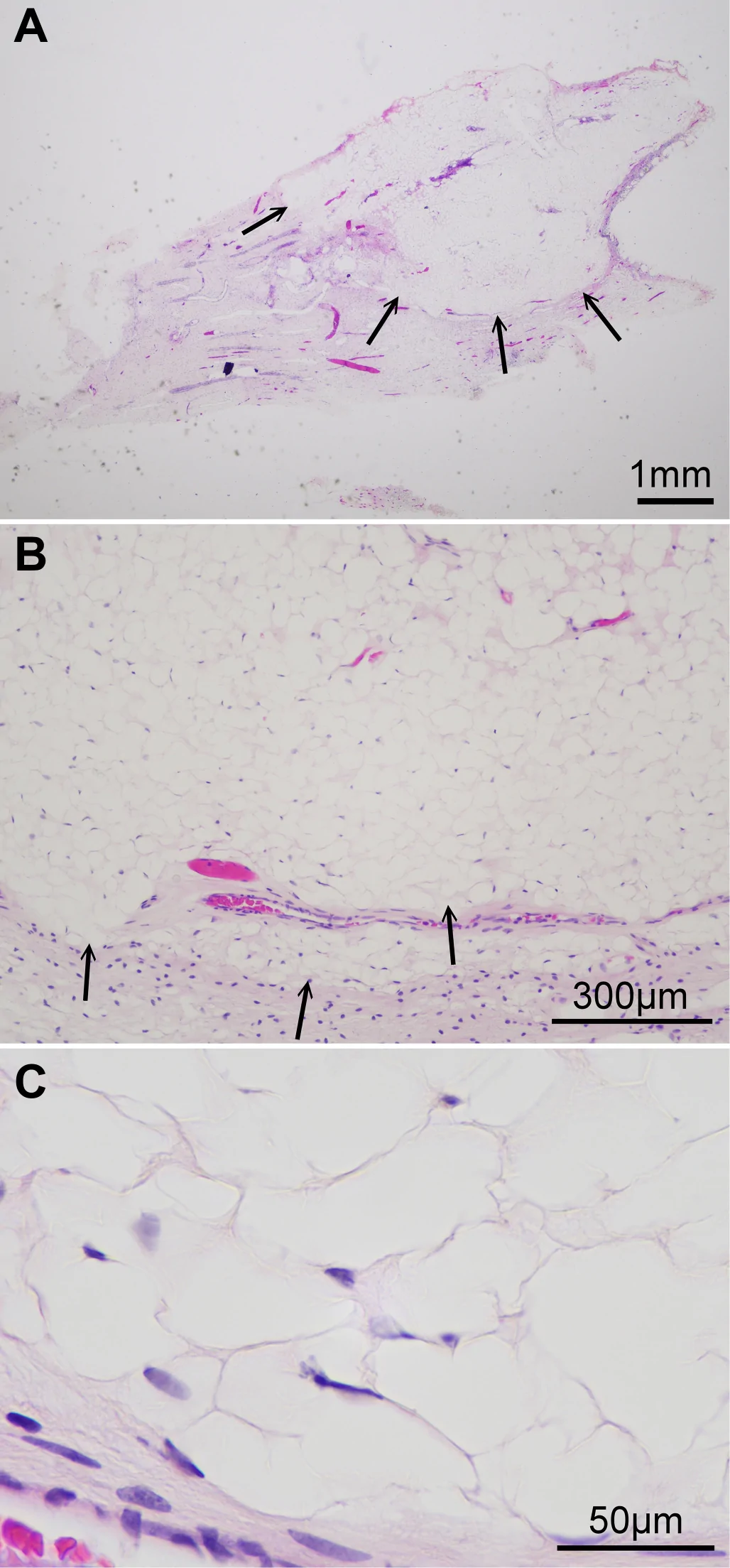
**Representative hematoxylin and eosin-stained histological sections of an upper second premolar with an International Caries Detection and Assessment System score of 3, showing extensive pulp degeneration (grade 3) in the same specimen.** (A) Low-magnification overview of the longitudinal pulp section, with arrows indicating multiple areas of degenerative change. (B) Higher-magnification view showing the transition between preserved pulp tissue and degenerative areas, characterized by vacuolated spaces, reduced cellularity, and disruption of normal pulp architecture (arrows). (C) High-magnification view of the degenerative region demonstrating prominent optically clear vacuolated spaces consistent with advanced pulp degeneration. Scale bars: 1 mm in (A), 300 µm in (B), and 50 µm in (C). **Abbreviation**: HE, hematoxylin and eosin.

### Area of PD

To provide a quantitative correlate to histological grading of pulpal degeneration and its relationship with MRI *T*_2_ relaxation times, we additionally assessed the proportion of pulp tissue exhibiting degenerative changes.

Under 10× magnification, the total cross-sectional area of each pulp specimen and the area demonstrating degenerative morphology were measured in mm^2^, and the percentage of affected pulp tissue was subsequently calculated.

### Statistical analysis

Mean *T*_2_ relaxation times and their variability were calculated for each ROI on the *T*_2_-weighted images. Statistical analyses were performed using SigmaPlot 14.0 (Systat Software, Inc., USA). Additional sensitivity analyses and bootstrap confidence interval estimations were performed using IBM SPSS Statistics (version 29, IBM Corp., Armonk, NY, USA). Normality assumptions were assessed using the Shapiro–Wilk test.

Comparisons among ICDAS groups for non-normally distributed variables were performed using the Kruskal–Wallis test, followed by Dunn’s post hoc test for multiple comparisons where appropriate. A significance threshold of *P <* 0.05 was applied for all statistical analyses.

Sample size estimation, based on a desired statistical power of 0.8 and a significance level of *P <* 0.05, indicated a minimum requirement of 19 specimens. The current study included 52 teeth, exceeding this threshold.

To examine the relationship between pulpal degeneration and MRI parameters, correlations between MRI-derived *T*_2_ mapping parameters and histological measures of PD were evaluated using Spearman’s rank correlation coefficient. Bootstrap-derived 95% confidence intervals (CIs) for Spearman correlation coefficients were calculated using 1,000 bootstrap samples. Statistical significance for correlations was defined at *P <* 0.05. Comparisons of mean *T*_2_ relaxation times and *T*_2_ variability between teeth with and without histologically confirmed PD were performed using independent-samples Student’s *t*-tests following assessment of normality (Shapiro–Wilk test) and homogeneity of variance (Brown–Forsythe test). Mean differences with corresponding 95% confidence intervals were calculated from the *t*-test analyses. Cohen’s d was calculated as a measure of effect size.

To assess the potential influence of patient-level clustering, an additional sensitivity analysis was performed using one representative tooth per participant. For participants contributing multiple teeth, the tooth with the highest ICDAS score was selected. If multiple teeth from the same participant shared the highest ICDAS score, the first available tooth in the dataset was selected. The principal correlation analyses were repeated using this patient-level dataset (*N* ═ 14).

## Results

A total of 52 permanent premolars (both upper and lower) were collected from 14 patients (9 females, 5 males) with a mean age of 14.2 ± 1.97 years.

### Caries severity assessment (ICDAS)

Of the 52 teeth examined, caries severity, assessed using the ICDAS, showed the following distribution: 15 teeth were intact (ICDAS 0, Group A), 7 teeth had initial enamel caries (ICDAS 1, Group B), 13 teeth had established enamel caries (ICDAS 2, Group C), and 17 teeth had dentin caries (ICDAS 3, Group D) (Individual data are provided in Table S1).

### PD in pulp tissue

Histological examination revealed PD in 12 of the 52 teeth.

### Histomorphological features of PD

Histologically, PD was characterized by areas of reticular atrophy, presenting as prominent empty or vacuolated spaces within the pulpal tissue, accompanied by a decrease in cellular density. These structural alterations are typically associated with degeneration and, in some regions, a loss of the odontoblastic layer [14]. Degenerated pulp tissue often resembled adipose-like structures, containing multiple optically clear, vacuolated spaces. These altered regions were distinctly demarcated from the adjacent healthy pulp tissue by a sharp and well-defined boundary (Figures 2 and 3).

**Figure 3.**
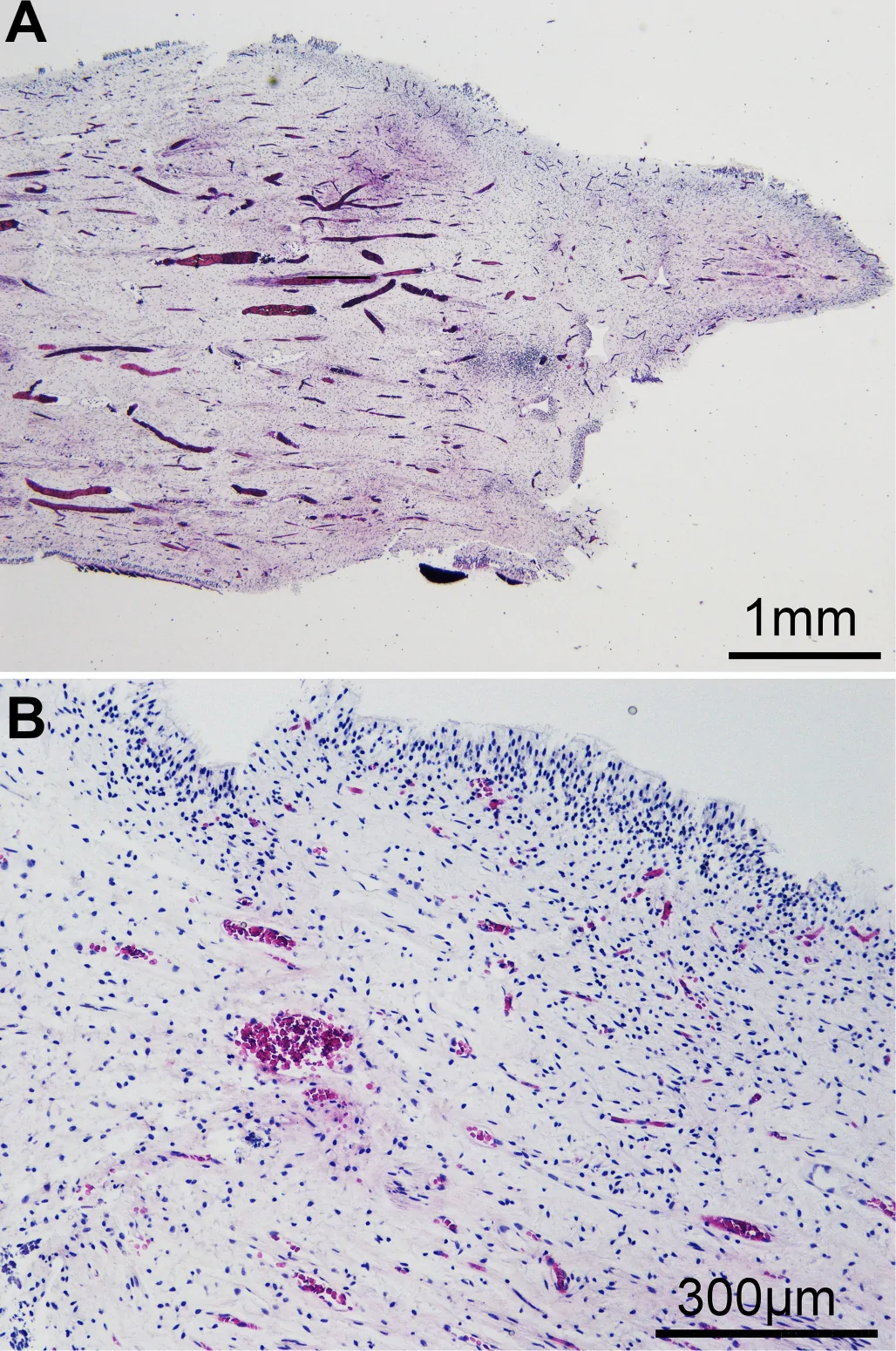
**Representative hematoxylin and eosin-stained histological sections of pulp tissue from a lower second premolar with an International Caries Detection and Assessment System score of 2, showing no detectable pulp degeneration (grade 0).** (A) Low-magnification overview of the longitudinal pulp section demonstrating preserved overall tissue organization. (B) Higher-magnification view showing intact pulp architecture, with preserved cellularity and no evidence of vacuolated or reticular degenerative changes. Scale bars: 1 mm in (A) and 300 µm in (B). **Abbreviation**: HE, hematoxylin and eosin.

### Degree of PD

PD was exclusively observed in carious teeth, with no PD detected in intact teeth (ICDAS 0) or those with an ICDAS score of 1. Among teeth with an ICDAS score of 2, four specimens exhibited PD, corresponding to grades 1 or 2 on a 0–3 scale. In teeth with an ICDAS score of 3, PD was present in eight specimens, with grades ranging from 1–3. These findings indicate that PD is associated with more advanced carious lesions and increases in severity with higher ICDAS scores (Table S2).

### Area of PD

The mean relative area of PD, calculated as the proportion of the PD area to the total histological section area in the 12 affected teeth, was 0.0682 (6.82%).

### MRI findings: *T*_2_ relaxation times

MRI-derived *T*_2_ relaxation times in the coronal pulp region were significantly lower in teeth with histologically confirmed PD compared to teeth without PD. Teeth without PD (*n* ═ 40) exhibited a mean *T*_2_ value of 131.740 ± 23.562 ms, while teeth with PD (*n* ═ 12) demonstrated a mean *T*_2_ value of 114.236 ± 28.020 ms. The mean difference between groups was 17.504 ms (95% CI: 0.049–34.959; *P ═* 0.049), corresponding to a Cohen’s d effect size of 0.68.

Similarly, the variability of MRI-derived *T*_2_ relaxation times in the coronal pulp region was lower in teeth with histologically confirmed PD. Teeth without PD (*n* ═ 40) exhibited a mean *T*_2_ variability of 41.463 ± 8.366 ms, whereas teeth with PD (*n* ═ 12) showed a mean *T*_2_ variability of 33.226 ± 7.080 ms. The mean difference between groups was 8.236 ms (95% CI: 2.435–14.038; *P ═* 0.006), corresponding to a Cohen’s d effect size of 1.06.

### Correlations between measured parameters

A significant positive correlation was observed between the grade of PD and the ICDAS score (*ρ ═* 0.481, 95% CI: 0.295–0.646; P ═ 0.00035). Conversely, a negative correlation was found between the grade of PD and the *T*_2_ relaxation times of dental pulp (*ρ ═* −0.367, 95% CI: −0.612 to −0.113; P ═ 0.00896). Additionally, negative correlations were observed between *T*_2_ relaxation time variability and the grade of PD (*ρ ═* −0.381, 95% CI: −0.659 to –0.197; P ═ 0.00708), between the area of PD and the *T*_2_ relaxation times (*P ═* −0.365, 95% CI: −0.610 to −0.116; P ═ 0.00934), and between the area of PD and *T*_2_ variability (*ρ ═* −0.379, 95% CI: −0.659 to −0.197; P ═ 0.00738).

Overall, greater histological PD consistently correlated with lower *T*_2_ relaxation times and *reduced T*_2_ variability, indicating that more advanced degenerative changes corresponded to decreased MRI-derived *T*_2_ values and signal heterogeneity.

### Sensitivity analysis

To evaluate the potential influence of patient-level clustering, the primary correlation analyses were reiterated using one representative tooth per participant (*N* ═ 14). For participants contributing multiple teeth, the tooth with the highest ICDAS score was selected. The observed associations remained consistent with those identified in the full tooth-level dataset. Significant negative correlations were observed between PD grade and *T*_2_ relaxation times (Spearman’s *ρ ═* −0.725, 95% CI: −0.890 to −0.279, *P ═* 0.003), as well as between the relative area of PD and *T*_2_ relaxation times (Spearman’s *ρ ═* −0.752, 95% CI: −0.932 to −0.277, *P ═* 0.002).

## Discussion

The current study investigates the intricate relationship among clinical caries severity, pulp tissue histology, and quantitative MRI findings by integrating ICDAS assessment, *T*_2_ mapping MRI, and histological grading of PD. The results consistently demonstrate significant associations between caries severity and both structural and compositional changes within the vital pulp, underscoring the biological relevance of PD.

A positive correlation was observed between the ICDAS score and the grade of PD. Teeth with higher ICDAS scores exhibited more advanced PD, indicating a direct association between caries severity and histological PD. This finding suggests a link between clinically detectable caries and microscopic changes within the pulp tissue, even in vital teeth. These results align with previous histological observations describing degenerative pulp alterations in response to chronic irritation, such as periodontal disease [14, 16]. Currently, the literature lacks histological studies specifically addressing pulp tissue changes induced by caries severity, as existing studies have predominantly examined intact teeth [16, 18, 19] or teeth with periodontal pathology [14, 16].

PD has been characterized in the literature as a disruption of the normal pulp connective tissue architecture, marked by a loosening of the collagen framework, expansion of intercellular spaces, and an increase in ground substance [16]. While some authors have suggested that such changes might be attributable to histological processing artifacts [19, 20], the present findings imply a potential biological relevance of these observations. However, given the exploratory design, the relatively small number of PD-positive specimens, and the inherent limitations of histological sampling, influences related to processing, sampling, or multifactorial causes cannot be entirely excluded. Furthermore, histological PD was observed only in a subset of ICDAS 2 and 3 teeth, indicating that degenerative changes were not uniformly present despite similar clinical caries severity.

### Comparison of PD grade and area across ICDAS scores

Comparisons of PD grade and PD area across ICDAS scores are presented in Table 1 and Table 2, respectively. As normality assumptions were not met (Shapiro–Wilk test, *P <* 0.05), intergroup comparisons for both PD grade and PD area were performed using the Kruskal–Wallis test. Statistically significant overall group differences were identified for PD grade (*H* ═ 12.495, *P ═* 0.006) and PD area (*H* ═ 12.313, *P ═* 0.006). However, post hoc pairwise comparisons using Dunn’s test did not reveal statistically significant differences between individual groups.

**Table 1:** PD grade by ICDAS score

**ICDAS score**	**n**	**Missing**	**Median**	**25th percentile**	**75th percentile**
0	15	0	0.000	0.000	0.000
1	7	0	0.000	0.000	0.000
2	13	0	0.000	0.000	1.000
3	17	0	0.000	0.000	2.000

Kruskal–Wallis test, *P ═* 0.006. Post hoc pairwise comparisons utilizing Dunn’s test did not reveal statistically significant differences between individual groups. **Abbreviations**: ICDAS, International Caries Detection and Assessment System; N, number of teeth; PD, pulp degeneration.

**Table 2:** Relative proportion of PD area by ICDAS score

**ICDAS score**	**n**	**Missing**	**Median**	**25th percentile**	**75th percentile**
0	15	0	0.000	0.000	0.000
1	7	0	0.000	0.000	0.000
2	13	0	0.000	0.000	0.0299
3	17	0	0.000	0.000	0.0555

Kruskal–Wallis test, *P ═* 0.006. Post hoc pairwise comparisons utilizing Dunn’s test did not reveal statistically significant differences between individual groups. **Abbreviations**: ICDAS, International Caries Detection and Assessment System; n, number of teeth; PD, pulp degeneration.

Quantitative MRI findings corroborate this interpretation. Both the degree and area of PD exhibited a negative correlation with *T*_2_ relaxation times, indicating that *T*_2_ relaxation times decreased as the severity of degeneration increased. Given the sensitivity of *T*_2_ relaxation times to tissue water content, this may suggest that PD is associated with reduced water accumulation within the pulp tissue. This interpretation aligns with histological descriptions of PD as a loose, porous, and consequently less hydrated tissue state.

Additionally, a negative correlation was observed between both the grade and area of PD and *T*_2_ relaxation time variability. As the severity of PD increased, *T*_2_ signal variability decreased, suggesting a more homogeneous pulp tissue composition. This reduced heterogeneity may reflect a loss of normal pulp microstructural organization as degenerative changes progress. Collectively, the observed changes in *T*_2_ relaxation times and variability provide complementary information regarding pulp tissue remodeling associated with caries [10].

Quantifying the percentage of pulp tissue affected by degeneration provided an objective morphometric parameter that complements semiquantitative histological grading and allows for a more direct comparison with MRI signal intensity and its variability. The calculated proportion of pulp area showing degenerative features varied among specimens and was negatively correlated with mean *T*_2_ relaxation times as well as *T*_2_ variability, indicating that greater histological degeneration was associated with lower *T*_2_ relaxation times.

Area-based quantification of histological features is a well-established method in pathology, frequently employed to determine the proportion of stained regions, necrotic zones, or other structural alterations within a given tissue section [21, 22]. By applying a similar morphometric strategy to dental pulp, this analysis provides an objective measurement of degenerative involvement that complements semiquantitative grading systems and facilitates correlation with imaging parameters.

However, a limitation of the present study stems from the inherent nature of histological methodology. The dental pulp is a three-dimensional structure with complex spatial organization, whereas our evaluation relied on two-dimensional sections obtained from single histological slides. Consequently, the extent of pulpal degeneration may have been either overestimated or underestimated. A single section might capture a relatively broad area of altered tissue while the actual defect is spatially limited, or conversely, only a small portion of a longitudinally extensive degenerative region may be represented in the examined plane. Therefore, these findings warrant cautious interpretation.

Furthermore, it is important to acknowledge that the pulpal degenerative features observed microscopically in this study may not be solely attributable to caries severity. All examined samples were premolars scheduled for orthodontic extraction, originating from dentitions characterized by crowding. Chronic mechanical stress, altered occlusal loading, and potential microvascular compromise associated with dental crowding may have contributed to the observed histological appearance of the pulp tissue [18, 23]. In this context, the observed changes may reflect a multifactorial process rather than a direct consequence of carious insult alone. Moreover, previous histological investigations have documented reticular atrophy and related degenerative patterns in clinically intact teeth, suggesting that such alterations can occur independently of carious pathology. These considerations collectively indicate that pulpal degeneration should be interpreted cautiously, recognizing that structural and environmental factors intrinsic to crowded dentitions may also play a role. While vacuolated or reticular pulp changes have historically been debated as potential histological artifacts [19], the reproducible morphology of the observed lesions and their associations with MRI-derived *T*_2_ mapping parameters and caries severity suggest their potential biological relevance [24].

Several other limitations of the present study warrant acknowledgment. Despite its exploratory design, the relatively limited sample size may restrict the precision and generalizability of the observed associations and group differences. Additionally, the associations between caries severity, histological PD, and MRI-derived *T*_2_ mapping parameters should be interpreted within the constraints of this cross-sectional study and do not establish direct causal relationships.

Another limitation is the absence of advanced carious lesions (ICDAS scores 4–6) in the examined sample. The included teeth were obtained from young individuals undergoing orthodontic treatment, thus precluding advanced dentinal cavitation or extensive structural breakdown. Consequently, the observed associations between MRI parameters and histological PD reflect only early to moderate stages of caries severity. Future studies incorporating teeth with more advanced lesions are necessary to determine whether the identified correlations persist across the full spectrum of caries severity.

## Conclusion

The integration of MRI with histological assessment in this study highlights the potential of quantitative MRI as a noninvasive approach for evaluating pulp tissue condition. Unlike conventional diagnostic methods, MRI enables direct assessment of soft tissue characteristics without ionizing radiation. The observed associations between MRI-derived *T*_2_ mapping parameters and histological findings support the relevance of MRI for investigating pulpal structural alterations associated with caries severity.

Histological PD was observed in a subset of teeth with higher ICDAS scores and demonstrated associations with quantitative MRI parameters. These findings suggest that MRI-derived *T*_2_ mapping may reflect structural changes within the dental pulp associated with caries severity. However, given the exploratory nature of this study and the limited sample size, further investigations involving larger cohorts and longitudinal study designs are necessary to better elucidate the biological and potential clinical significance of these findings.

## Supplemental data

**Table S1:** Tooth-level histological assessment of PD

**Specimen ID**	**Tooth**	**ICDAS score**	**PD present**	**PD grade**	**Relative degeneration area (PD/Section)**
Z1	14	0	No	0	0
Z1	24	0	No	0	0
Z1	34	0	No	0	0
Z1	44	0	No	0	0
Z2	24	0	No	0	0
Z2	34	0	No	0	0
Z3	15	3	Yes	1–2	0.00786
Z3	25	3	Yes	3	0.32159
Z3	35	3	No	0	0
Z3	45	3	No	0	0
Z4	14	3	Yes	2	0.08839
Z4	24	3	Yes	2	0.04308
Z4	35	3	Yes	1–2	0.03392
Z4	45	3	Yes	1	0.02317
Z5	15	3	No	0	0
Z5	24	3	No	0	0
Z5	34	2	No	0	0
Z5	44	2	No	0	0
Z6	14	2	No	0	0
Z6	24	3	Yes	3	0.08698
Z6	34	1	No	0	0
Z6	44	1	No	0	0
Z7	15	3	No	0	0
Z7	25	2	No	0	0
Z7	35	3	No	0	0
Z7	45	2	No	0	0
Z8	14	1	No	0	0
Z8	24	1	No	0	0
Z8	34	1	No	0	0
Z8	44	0	No	0	0
Z9	14	2	Yes	2	0.052307
Z9	24	2	Yes	1	0.018726
Z9	34	2	No	0	0
Z9	44	2	Yes	1	0.041121
Z10	14	2	Yes	2	0.044427
Z10	24	2	No	0	0
Z10	34	1	No	0	0
Z10	44	1	No	0	0
Z11	14	2	No	0	0
Z11	24	2	No	0	0
Z12	14	0	No	0	0
Z12	24	0	No	0	0
Z12	34	0	No	0	0
Z12	44	0	No	0	0
Z13	15	0	No	0	0
Z13	25	0	No	0	0
Z13	35	0	No	0	0
Z13	45	0	No	0	0
Z14	14	3	No	0	0
Z14	24	3	Yes	2	0.067867
Z14	34	3	No	0	0
Z14	44	3	No	0	0

Intermediate grades were coded as continuous values for statistical analyses (e.g., 1–2 = 1.5; 2–3 = 2.5). **Abbreviations**: ICDAS, International Caries Detection and Assessment System; ID, identification; PD, pulp degeneration.

**Table S2:** Distribution of histological pulp degeneration grades by ICDAS score

**ICDAS score**	**PD grade 0**	**PD grade 1**	**PD grade 1–2**	**PD grade 2**	**PD grade 2–3**	**PD grade 3**	**Total**
0	15	0	0	0	0	0	**15**
1	7	0	0	0	0	0	**7**
2	9	2	0	2	0	0	**13**
3	9	1	2	3	0	2	**17**
**Total**	**40**	**3**	**2**	**5**	**0**	**2**	**52**

**Abbreviations:** ICDAS, International Caries Detection and Assessment System; PD, pulp degeneration.
